# Biomarkers to predict the severity of acute pancreatitis

**DOI:** 10.3389/fmed.2025.1619087

**Published:** 2025-08-06

**Authors:** Xinwei Hao, Zimeng Wang, Xiaotong Niu, Longsong Li, Yawei Bi, Ningli Chai

**Affiliations:** Department of Gastroenterology, The First Medical Center of Chinese PLA General Hospital, Beijing, China

**Keywords:** acute pancreatitis, biomarkers, extracellular traps, intestinal flora, mitochondrial DNA

## Abstract

Acute pancreatitis (AP) is pancreatic inflammation caused by abnormal activation of trypsinogen, and moderately severe or severe acute pancreatitis (SAP) can lead to systemic inflammatory response syndrome (SIRS) and organ failure, associated with high mortality. Therefore, early prediction of the severity of acute pancreatitis is particularly important to improve patient survival rate and reduce complications. Currently, many scoring systems (e.g., Ranson scoring, etc) and classical biomarkers are available in the clinical practice, but there are still many limitations, such as low predictive value and time delay. Potential biomarkers for the prediction of SAP are still a hot topic in current research. In this review, we aim to summarize newly discovered biomarkers for the prediction of the severity of acute pancreatitis in the recent years, and provide an overview of serum markers, intestinal flora, and genetic markers. At the same time, the development of emerging detection technologies [e.g., Robust AP Identification and Diagnosis (RAPIDx) and droplet digital PCR (ddPCR)] also provides new possibilities for early prediction of SAP, allowing these biomarkers to be applied clinically.

## Introduction

Acute pancreatitis (AP) is a rapidly developing inflammatory process of the pancreas and is one of the most common diseases of the digestive system (1). Most patients have only mild acute pancreatitis (MAP), with symptoms resolving within 1 week. About 20% of patients develop moderately severe or severe acute pancreatitis (SAP) with pancreatic necrosis and even organ failure (2, 3). Acute pancreatitis is featured by damage to the alveolar cells, which prompts inappropriate release and activation of trypsinogen. This triggers the activation of other digestive enzymes, the kinin system and the complement cascade, leading to the self-digestion of the pancreatic parenchyma and even systemic inflammatory response syndrome (SIRS) (4). Revision of the Atlanta classification in 2012 (5) categorizes acute pancreatitis into three degrees of severity: (1) mild acute pancreatitis (MAP): absence of organ failure and local or systemic complications, with early recovery and a very low mortality rate; (2) moderately severe acute pancreatitis: the presence of transient organ failure (<48 h) or local or systemic complications in the absence of persistent organ failure; and (3) severe acute pancreatitis (SAP): the presence of persistent organ failure (>48 h), and the development of infected necrosis among patients with persistent organ failure is associated with an extremely high mortality (20%−40%). Given the variable clinical course in acute pancreatitis and the significant mortality rate in severe cases, early prediction of SAP has become a very important clinical issue. Scoring systems such as the Ranson score, the Acute Physiology and Chronic Health Evaluation (APACHE) II score, Bedside Index of Severe Acute Pancreatitis, the Computed Tomography Severity Index, and classical serologic molecules have emerged to predict the severity of AP (4, 6, 7). Potential biomarkers for the prediction of SAP are still a hot topic in current research. In recent years, researches targeting the pathophysiological mechanisms of acute pancreatitis have identified a large number of emerging biomarkers. This article summarizes some emerging biomarkers, including serum markers, intestinal flora and genetic markers. At the same time, by analyzing the pathogenesis of AP, we find a potential molecule that may play a role in predicting SAP in the hope that it can be helpful in the subsequent clinical applications.

## Serum markers

### Serum amyloid A (SAA) protein

Serum amyloid A (SAA) protein is an early and sensitive biomarker of inflammatory diseases, with expression up-regulated 1,000-fold during inflammation, infection and tissue injury (8, 9). Increased transcription of the A-SAA gene in the acute phase response was demonstrated both *in vivo* and *in vitro* (10). In a caerulein-induced AP animal model, the expression of the specific SAA isoform, SAA3, was found to be significantly elevated. Compared with wild-type mice after administration of caerulein, the lower levels of serum amylase and lipase, milder tissue damage, and less production of pro-inflammatory cytokines in the pancreas were observed in SAA3 knockout (Saa3^−/−^) mice, suggesting a correlation between SAA and the severity of AP (11). As a biomarker of predicting AP severity detected at an early stage, SAA had a better performance than CRP in determining the severity of AP (10, 12). A multicenter prospective study that included 246 patients showed that SAA was superior to CRP in predicting the severity of pancreatitis on admission (area under curve 0.7 vs. 0.59, respectively; *P* = 0.02) and 24 h or less after the onset of symptoms (12). The commercially available kits for A-SAA detection in clinical laboratories are mainly based on immunoturbidimetric or immunonephelometric technology. But most of them are polyclonal antibody-based assays that cannot distinguish among the different A-SAA isotypes (10). It is worth mentioning that SSA proteins on extracellular vesicles (EVs) are also potential biomarkers for differential expression in patients with AP of varying severity. Recently a rapid and high-sensitive detection method, Robust AP Identification and Diagnosis (RAPIDx) method was constructed by proteomic fingerprinting of intact nanoscale extracellular vesicles. The RAPIDx allows a fast EV isolation step from plasma samples via EXODUS within 15 min followed by high-throughput MALDI-TOF-MS detection of AP in 1 min. By using this method to analyze the EVs of 115 clinical samples, the SAA proteins was demonstrated to be a promising biomarker to discriminate AP severity with an AUC of 83% (13).

### Neutrophil extracellular trap

Neutrophils play a role in the progression of acute pancreatitis (AP). In addition to secreting antimicrobial compounds, activated neutrophils can also form extracellular meshwork structures known as neutrophil extracellular traps (NETs) through the expulsion of nuclear DNA and histone proteins. It has been demonstrated that neutrophil-derived NETs constitute a central component in the pathophysiology of severe AP and the plasma levels of NET components increased in patients with severe AP (14).

The potential role of NETs in the tissue damage in AP has been reported in numerous studies. NETs promote neutrophils infiltration which is a key component in AP, which can activate neutrophils directly or indirectly by upregulating Mac-1 expression on neutrophils. Mac-1 is an adhesion molecule that promotes extravascular accumulation of neutrophils at sites of inflammation. Moreover, NETs regulate signal transducer and activator of transcription 3 (STAT3) activity in acinar cells and promote trypsin activation in acinar cells through MMP-9 (14), exacerbating the degree of pancreatic tissue damage. A recent study showed that the formation of NETs in AP requires the participation of the myeloperoxidase–neutrophil elastase pathway, and the citrullination of histone H3 in SAP is affected by the activity of NE (15). In a mice model, Linders et al. (16) demonstrated that C3 induces neutrophil recruitment and the formation of neutrophil extracellular traps. A recent study explored the mechanism of the highly expressed P-selectin stimulating NETs formation in AP. They found that the levels of NETs and P-selectin in patients with AP was higher than that in healthy volunteers. And P-selectin induces NETs formation in neutrophils through PSGL-1 and its downstream Syk/Ca^2+^/PAD4 signaling pathway. Inhibiting p-selectin blunted NETs formation and ameliorated the severity of AP in mice (17, 18).

The quantitative determination of NETs was achieved by measuring cell free DNA (cf-DNA) levels or the concentration of MPO-DNA complexes, which limits the clinical application of NETs. Nowadays, a novel and comprehensive flow cytometry approach for the measurement of circulating cell appendant NETs has been developed (19). It is hoped that more research will focus on the application of this method in the early diagnosis of severe acute pancreatitis. At the same time, the discovery of these molecular pathways provides us with ideas for finding potential biomarkers in the future. The high sensitivity and specificity of neutrophil elastase (20) and complement C3 (21–23) in predicting SAP has been demonstrated, and we may be able to look for new biomarkers in these potential molecular pathways that may be serve as determining AP severity.

### Extracellular vesicle

Extracellular vesicles (EVs) are nanoscale bioparticles that transport biomolecules (RNA, proteins, and metabolites) from disease-related organs or tissues to target cells for intercellular communication, mainly including exosomes (30–150 nm) and microvesicles (150–1,000 nm) (24, 25). Extracellular vesicles have been shown to be involved in the progression and exacerbation of many diseases, and are closely related to disease severity (26). Based on this characterization, EVs is a potential source for the discovery of new biomarkers. In acute pancreatitis, EVs can be directly released into peripheral blood circulation from the inflammatory pancreas in the early stages of the disease, carrying information relevant to disease progression before cell necrosis (27, 28). An efficient exosome detection method via the ultrafast-isolation system, EXODUS, has been established to achieve the isolation of EVs with high purity and yield from the plasma (29). This also laid the foundation for subsequent high-throughput detection. In addition to the SAA proteins described above, there are many other biomarkers in EVs that play a role in predicting the severity of acute pancreatitis. Four biomarkers for SAP prediction were identified from plasma EVs by EXODUS system and quantitative metabolomic analyses, including eicosatrienoic acid (C20:3), thiamine triphosphate, 2-acetylfuran, and cis-citral. The area under the curve (AUC) of the panel was >0.95 for both discovery (*n* = 30) and validation (*n* = 70) sets (27).

A comprehensive transcriptomic and metabolomic analysis of sEVs revealed that macrophage migration inhibitory factor and tubulin alpha 1b may be key variables that play an important role in AP progression, serving as potential markers for the diagnosis of SAP (30, 31). Increased amounts of S100A8 and S100A9 carried by extracellular vesicles in severe acute pancreatitis have been reported to activate NADPH oxidase to produce free radicals that promote inflammatory responses and exacerbate pancreatic damage (32).

### Others

The progression of AP is closely related to immune function. Overactivation of inflammatory cells and their cytokines is one of the pathological mechanism of AP (33). Some immune-related molecules may be potential markers for predicting the severity of acute pancreatitis. T cell immunoglobulin and mucin domain-3 (TIM-3) is initially founded to be expressed in activated Th1 cells, and it has been thought to play a crucial role in the negative regulation of immune responses through interactions with its ligand galectin-9 (34, 35). In a retrospective study, sTIM-3 levels were detected by ELASA kits and proved to have good predictive efficacy with an AUC of 0.853 (34). Circulating extracellular nicotinamide phosphoribosyl transferase (eNAMPT) exerts the role of a cytokine regulator of innate immunity by binding to Toll-Like receptor 4 and nuclear factor-κB (NF-κB) activation. Pancreatitis circulating eNAMPT levels are significantly elevated in acute pancreatitis, and have positive correlation with disease severity (36). In addition, soluble suppression of tumorigenicity 2 (sST2) protein (37) regulates the function and differentiation of IL-33/ST2-mediated Th1 and Th2 Lymphocytes in AP homeostasis. And the optimal cut-off value of serum sST2 levels detected by ELASA kits as an indicator for prediction of SAP was projected to be 1,190 pg/ml, with the area under the curve 0.889. These biomarkers have been shown to be useful in clinical trials in predicting the severity of acute pancreatitis (Table 1).

**Table 1:** Biomarkers in predicting the severity of acute pancreatitis and predictive accuracy.

**Biomarkers**	**Cut-off level**	**Timing**	**Detection method**	**AUC**
Serum amyloid A (SAA) proteins (12)	418 mg/L	On admission	ELISA kit	0.70
SSA1-1 proteins on EVs (13)	/	/	RAPIDx	0.70
Neutrophil elastase (20)	110 μg/L	Within 72 h after symptom onset	Latex immunoagglutination	0.956
Complement C3 (23)	0.4 g/L	Within 24 h after symptom onset	EFSIA kit	0.749
The panel of C20:3, TTP, 2-AF, and cis-citral on EVs (27)	/	On admission	EXODUS + metabolomic analyses	0.935
sTIM-3 (34)	1.4 μg/L	Within 48 h after symptom onset	ELISA kit	0.853
sST2 (37)	1.19 μg/L	Within 24 h after symptom onset	ELASA kit	0.889

TTP, thiamine triphosphate; 2-AF, 2-acetylfuran; sTIM-3, T cell immunoglobulin and mucin domain-3; sST2, soluble suppression of tumorigenicity 2.

A number of emerging serum markers have been identified based on the pathogenesis of SAP, including von Willebrand factor (38, 39), soluble mannose receptor (sCD206) (38), Plasma Osteopontin (40). These biomarkers provide new ideas for our future experiment, and large, prospective clinical trials are needed to further validate their predictive efficacy. Basic medicine experiments are also needed to determine potential molecular pathways of these molecular markers in predicting the severity of AP. And efficient and rapid detection methods are anticipated to achieve early identification of SAP, to be able to apply these biomarkers to the clinic as early as possible. Recently, developments in nanotechnology have improved the sensitivity of serologic diagnosis of AP and have provided a new modality for the treatment of acute pancreatitis (41).

## Intestinal flora

The gut microbiota plays an important role not only in intestinal structure and function, but also in intestinal-associated immune system and epithelial cell function. More and more studies have shown that alterations in gut micro-ecology are associated with the progression of AP, including microbiota dysbiosis, intestinal barrier damage and immune dysfunction (42). Translocation of intestinal bacteria and endotoxins after intestinal barrier damage is the main cause for superinfection of pancreatic necrosis, which aggravated the severity of acute pancreatitis (43).

Multiple studies found that the intestinal flora have changed in SAP patients or mice models (Table 2). It has been demonstrated that the abundance of *Enterococcus* was significantly increased, and that of *Bifidobacterium* and *Blautia* was significantly decreased in patients with SAP (44, 45). Differential analysis of gut microbiota composition and functional enrichment was performed, and it found that representative pathways associated with the amino acid metabolism (valine, leucine, and isoleucine degradation) were enriched in SAP (45). Li et al. (46) has also demonstrated that hyper-triglyceridemic pancreatitis (HTGP) patients showed increased abundances of *Escherichia Shigella* and *Enterococcus*, and decreased abundances of *Bacteroides* and *Faecalibacterium*. The downregulated abundances of *Faecalibacterium prausnitzii* and *Bacteroides uniformis* indicated severe complications and poor outcome. The incidence of (HTGP) has been increasing in recent years, accounting for ~20%−30% of patients with acute pancreatitis. HTGP tends to have high incidence of complications and poor clinical prognosis, such as infected pancreatic necrosis and organ failure. Mice with long-term deficiency of Paneth cells exhibit more severe pancreatic pathological injuries and inflammation. The underlying mechanism may be intestinal flora dysbiosis and weakened intestinal mucosal barrier function. Mice lacking Paneth cells are accompanied by increases in *Enterococcus* and a decrease in *Bifidobacterium* (47, 48). And at the same time, the lack of antimicrobial peptides (AMPs) secreted by Paneth cells makes the intestinal barrier function reduced, leading to bacterial translocation (49). Restoring of partial Paneth cell function by supplementing lysozyme reduces the severity of acute pancreatitis and intestinal flora dysbiosis. These studies further confirmed the role that intestinal flora imbalance played in exacerbating pancreatic injury.

**Table 2:** Gut microbiota dysbiosis in severe acute pancreatitis.

**Studies**	**Source**	**Timing**	**Sample**	**Detection method**	**Intestinal flora**
Zou 2022 (76)	19 NP and 39 non-NP	Within 24 h after symptom onset	Rectal swabs	16S rRNA sequencing	*Enterococcus faecium*↑; *Finegoldia magna*↑
Ammer-Herrmenau 2024 (77)	28 SAP and 53 non-SAP in matched cohorts	Within 72 h of hospital admission	Buccal and rectal swabs	16S rRNA and metagenomic sequencing	*Parabacteroides distasonis*↑, *Roseburia hominis*↑, *Enterocloster bolteae*↑, *Dysosmobacter welbionis*↑; *Bifidobacterium adolescentis*↓, *Ligilactobacillus johnsonii*↓ etc.
Wang 2024 (78)	32 MAP and 17 SAP	Within 72 h after symptom onset	Fecal samples	16S rDNA sequencing	*Proteobacteria*↑, *Fusobacteriota*↑, *Actinobacteria*↑, *Desulfobacterota*↑, *Euryarchaeota*↑, *Nitrospirota*↑, *Verrucomicrobiota*↓
Liu 2024 (79)	25 SAP, 57 non-SAP and 115 HC	Within 72 h after symptom onset	Fecal samples	Whole-metagenome shotgun sequencing	*Escherichia coli*↑, *Parabacteroides distasonis*↑, *Enterococcus faecalis*↑; *Streptococcus salivarius*↓, *Streptococcus thermophilus*↓, etc.
Glaubitz 2023 (80)	Mice	/	Duodenal aspirates	16S rRNA sequencing	*Escherichia/Shigella*↑, *Enterococcus*↑, *Staphylococcus*↑; *Clostridia*↓, *Bacteroidetes*↓

NP, necrotizing pancreatitis. The arrows indicate the alteration of gut microbiota in AP compared with controls.

The severity of AP is also related to the metabolites of intestinal flora. Taurine plays an important role in maintaining neutrophil redox homeostasis and inhibiting NETs formation. As previously mentioned, NETs exacerbate pancreatic injury and systemic inflammation. The decreased abundance of *Bacteroides* in gut microbiota impairs taurine production. At the same time, it has been found that the taurine level in the serum decreases in severe HTGP patients. The mechanism is that taurine limits the activations of IL-17 and NF-κB signaling pathways in the neutrophils that repress NETs and pancreatic injury in HTGP (46). Short-chain fatty acids, one of the metabolites of intestinal microflora, have been confirmed to be reduced in AP patients (50). Reviews have described in detail the role of SCFA in AP (51). SCFA protects the intestinal barrier by regulating the expression and distribution of tight junction proteins and promoting the secretion of mucins on the intestinal surface (52). During the process of AP, dysbiosis of intestinal flora, SCFA reduction and intestinal barrier damage further aggravate pancreatic damage and promote the progression of AP. And the SCFA-producing bacterial flora and SCFA levels of SAP patients were significantly reduced compared with those of MAP patients (50). In a recent study, a total of three immunogenic cell death-related HUB genes (LY96, BCL2, and IFNGR1) were identified in SAP, and single-sample gene set enrichment analysis showed that the HUB genes were closely associated with the infiltration of specific immune cells, the activation of immune pathways, and the metabolism of single-chain fatty acids (especially butyric acid) (53), further confirming the importance of SCFA in identifying SAP.

## Genetic markers

### Mitochondrial DNA (mtDNA)

The loss of cell membrane integrity and release of intracellular contents gives necrotic cells the ability to induce inflammatory response. These immunogenic endogenous molecules are collectively called “damage-associated molecular patterns” (DAMPs) (54). In acute pancreatitis, dead pancreatic acini release intracellular damage-associated molecular patterns (DAMPs) including mtDNA, leading to the activation of various inflammatory signaling pathways [such as nuclear factor-κB (NF-κB), mitogen-activated protein kinase, signal transducer and activator of transcription 3 (STAT3), and inflammasome) and subsequent systemic inflammatory response syndrome, ultimately leading to organ damage in moderate to severe AP (55, 56). Unmethylated CpG regions in mtDNA make mtDNA resemble microbial DNA, which results in mitochondrial DNA being more immunogenic (57). At the same time, mtDNA can bind to pattern recognition receptors, thereby inducing the production of inflammatory cytokines (58). Yakah et al. (59) used a newly developed highly sensitive double droplet digital PCR (ddPCR), which achieves the absolute concentration of the mtDNA template (copy numbers/μl of plasma), to measure circulating plasma mtDNA fragments [D-Loop (DL), NADH ubiquinone oxidoreductase chain 1, and averaged mtDNA). The three types of plasma mtDNA fragments measured within 24 h of admission showed that the copy number of fragments in moderate severe or severe AP was higher than that in mild AP, and the difference was statistically significant. Additionally, receiver-operating characteristic curve analysis to distinguish AP severity yielded an area under the curve for mtDNA fragments of 0.91. An optimal cutoff value of 3.90, 5.62, and 4.76 copies/ml for DL, ND1, and averaged mtDNA fragments has been determined at a sensitivity of 0.90 and specificity of 0.90. Ezzat et al. (60) also found the familiar result.

### Others

Cell free DNA (cfDNA) is derived from genomic DNA released during cell death (apoptosis or necrosis) and, therefore carries cell type-specific epigenetic features from its source tissue (61). A SAP prediction model based on cfDNA methylation levels has been established (62). However, the measurement of methylation levels takes 2–3 days to complete, which limits the application of this biomarker in early diagnosis, but it still provides a new idea for our future experiments. And we can conduct organ/tissue specific research in cfDNA methylation levels to achieve molecular diagnosis of specific organ damage.

MicroRNAs play an essential role in the occurrence and progression of acute pancreatitis. Several recently published articles have elaborated on the potential mechanisms and biomarkers of MicroRNAs in the progression of SAP (63–65). Studies have also found three types of differentially expressed exosomal miRNAs in patients with SAP. The complement component 3 (C3) gene is the target gene of one of the differentially expressed miRNAs, which also indicates that C3 may serve as an early biomarker of SAP (21).

## Future perspectives

The development of bioinformatics and multi-omics analysis allows us to discover differentially expressed genes and proteins in SAP patients, but basic medical experiments are needed for validation. Moreover, researches on the molecular pathways and mechanisms of SAP can also help us predict emerging molecular markers. Early detection of biomarkers for severe acute pancreatitis will remain a problem we need to strive in the future, in order to achieve early clinical detection and timely intervention of SAP patients.

In searching the literature, we found that receptor-interacting protein kinase 1 (RIPK1) plays an important role in multiple inflammatory diseases (66–68). RIPK1 is a major mediator of multiple cell death pathways, including apoptosis and necroptosis, and inflammatory responses (69). The assembly and activation of multiple necrotic mechanisms, including RIPK1, are associated with NETosis, which in turn leads to the formation of neutrophil extracellular traps (NETs) (70). Activation of RIPK1 has been shown to promote the production of pro-inflammatory cytokines, including IL-6 and TNF, and inhibiting RIPK1 can block the occurrence of TNF-induced sepsis in animal models (71, 72). These mechanisms have been confirmed to play an important role in the pathophysiology of acute severe pancreatitis.

The *in vivo* results showed that inhibition of RIPK1 expression significantly reduced the extent of pancreatic tissue necrosis, inhibited the massive release of inflammatory mediators and oxidative stress damage, and ultimately effectively decreased the severity of AP (73). This process might be potentially regulated by the RIPK1/NF-κB/AQP8 pathway. Yao et al. (74) has founded that dexmedetomidine (Dex) attenuated SAP-induced pancreatic injury, infiltration of neutrophils and macrophages, and oxidative stress. Further mechanisms suggest that Dex inhibited the expression of necroptosis-associated proteins RIPK1, RIPK3, and MLKL and alleviated apoptosis in acinar cells (74). And disulfiram could also moderate the severity of mouse acute pancreatitis by inhibiting RIPK-dependent acinar cell necrosis and the following formation of NETs (75).

Serum levels of RIPK1 can be determined by ELISA kit. It has been demonstrated that serum RIPK1 levels were correlated positively with the severity of bulbar symptoms in the patients with amyotrophic lateral sclerosis, which can be used as clinical biomarker for the activation of RIPK1 in central nervous system (69). However, the level of serum RIPK1 is not currently used to predict the severity of acute pancreatitis. Further studies on the potential of RIPK1 as an emerging biomarker are still required.

## Conclusion

Early identification of SAP will help us take more aggressive treatment measures to improve patient survival rates and reduce the occurrence complications. Currently, early prediction methods of the severity of acute pancreatitis include clinical scoring systems, inflammatory markers, and imaging assessments. The Ranson scoring, the APACHE II scoring and the bedside severity index scoring system are more widely used in clinical practice, but still have some limitations. Biomarkers of SAP has become a hot research topic since the last century, including some classic molecules, such as IL-6, CRP, etc. With the development of assay technologies, early, rapid, and sensitive detection of classical serum biomarkers has been achieved. In addition, the intestinal flora may play an important role in the development of SAP since most bacteria causing necrotic infection of pancreatic tissue are from the intestinal flora. Moreover, dead pancreatic acini release mtDNA leading to the activation of various inflammatory signaling pathways and subsequent systemic inflammatory response syndrome, ultimately leading to organ damage in moderate to severe AP. This review summarizes its predictive role in severe acute pancreatitis from the above three aspects, looking forward to early clinical application.
